# Traditional Nonsteroidal Anti-Inflammatory Drugs and Postmenopausal Hormone Therapy: A Drug–Drug Interaction?

**DOI:** 10.1371/journal.pmed.0040157

**Published:** 2007-05-22

**Authors:** Luis Alberto García Rodríguez, Karine Egan, Garret A FitzGerald

**Affiliations:** 1 Centro Español de Investigación Farmacoepidemiológica, Madrid, Spain; 2 The Institute for Translational Medicine and Therapeutics, University of Pennsylvania, Philadelphia, Pennsylvania, United States of America; University of California San Francisco, United States of America

## Abstract

**Background:**

Suppression of prostacyclin (PGI_2_) is implicated in the cardiovascular hazard from inhibitors of cyclooxygenase (COX)-2. Furthermore, estrogen confers atheroprotection via COX-2–dependent PGI_2_ in mice, raising the possibility that COX inhibitors may undermine the cardioprotection, suggested by observational studies, of endogenous or exogenous estrogens.

**Methods and Findings:**

To identify an interaction between hormone therapy (HT) and COX inhibition, we measured a priori the association between concomitant nonsteroidal anti-inflammatory drugs (NSAIDs), excluding aspirin, in peri- and postmenopausal women on HT and the incidence of myocardial infarction (MI) in a population-based epidemiological study. The odds ratio (OR) of MI in 1,673 individuals and 7,005 controls was increased from 0.66 (95% confidence interval [CI] 0.50–0.88) when taking HT in the absence of traditional (t)NSAIDs to 1.50 (95% CI 0.85–2.64) when taking the combination of HT and tNSAIDs, resulting in a significant (*p* < 0.002) interaction. The OR when taking aspirin at doses of 150 mg/d or more was 1.41 (95% CI 0.47–4.22). However, a similar interaction was not observed with other commonly used drugs, including lower doses of aspirin, which target preferentially COX-1.

**Conclusions:**

Whether estrogens confer cardioprotection remains controversial. Such a benefit was observed only in perimenopausal women in the only large randomized trial designed to address this issue. Should such a benefit exist, these results raise the possibility that COX inhibitors may undermine the cardioprotective effects of HT.

## Abstract 2

It is controversial whether estrogens confer cardioprotection. This study suggests that even should such a benefit exist, COX inhibitors may undermine cardioprotective effects of hormone therapy.

## Editors' Summary

**Background.:**

There is currently a great deal of uncertainty regarding the effect of postmenopausal hormone therapy on heart disease in women. Premenopausal women are much less likely to experience heart attacks and strokes than men, a difference that does not exist between postmenopausal women and men. One mechanism that might explain these observations relates to the effect of estrogen, which is thought to have a protective effect on the heart. Hormone replacement therapy (HT) consisting of replacement estrogen, and sometimes progesterone as well, is often taken by women experiencing symptoms of menopause. Evidence from observational studies and the Womens' Health Initiative (WHI) trial has suggested that HT protects against heart disease in perimenopausal women. However, researchers have suggested that any beneficial effect of hormone replacement therapy on the heart might be counteracted by the effects of certain types of painkillers also being taken by women involved in the studies. These painkillers, nonsteroidal anti inflammatory drugs ( NSAIDs), prevent production of a molecule called prostacyclin. Prostacyclin plays a role in preventing blood clotting and is therefore thought to be important in protecting the heart. Estrogen, however, acts to increase production of prostacyclin, and it is therefore theoretically possible that hormone replacement therapy does have a beneficial effect on heart health, but which is counteracted by the negative effects of NSAIDs.

**Why Was This Study Done?:**

In this study, the researchers wanted to find out whether there was any evidence for an interaction between NSAID use, hormone replacement therapy, and heart disease. Such understanding in turn might help to identify more clearly whether hormone replacement therapy protects against heart disease in specific subgroups of postmenopausal women.

**What Did the Researchers Do and Find?:**

This study was carried out using information from the UK's General Practice Research Database, which is the largest computer database of anonymous medical records from primary care anywhere in the world. It contains information entered by UK general practitioners on their patients' drug prescriptions, diagnoses, referrals to hospital, and other data. The researchers here searched for all individuals from the database who were aged between 50 and 84 years on 1 January 1997, and then followed them up through the database for four years, or until the individual died, reached 85 years of age, or was diagnosed with a heart attack or cancer. From this search, the researchers found 1,673 women who had heart attacks or who died from coronary heart disease; these were considered “cases.” Then, these 1,673 women were matched against 20,000 “control” women of similar age. Information was pulled out for each case or control on their use of hormone replacement therapy, NSAIDs (covering 21 different drugs, but most commonly diclofenac, ibuprofen, and naproxen), and various risk factors for heart disease. The researchers then compared use of hormone replacement therapy and NSAIDs between the cases and controls, while making statistical adjustments for other risk factors (such as diabetes and smoking, for example).

The researchers found that current use of hormone replacement therapy was associated with a lower risk of heart attack than non-use. The odds ratio (chance of a heart attack among HT users compared to the chance among non-users of HT) was 0.78. However, when looking at women who used NSAIDs at the same time as hormone replacement therapy, the researchers found no suggestion of a reduction in risk of heart attack: the odds ratio for the chance of heart attack among this group of women, as compared to nonusers of both NSAIDs and hormone replacement therapy, was 1.50.

**What Do These Findings Mean?:**

These findings suggest that hormone replacement therapy and NSAIDs might interact, with NSAIDs acting against a role for hormone replacement therapy in preventing heart attacks. At face value, these results are in conflict with the findings of one large trial, the WHI trial, which failed to find a benefit of HT in preventing heart attacks. However, a recent analysis of WHI suggests cardioprotective effects of HT in women close to the time of the menopause and this coincides with the younger age of women in the observational studies such as the present one rather than in the WHI overall. Observational research studies, such as the present one, are often difficult to interpret because the groups being compared are not necessarily equivalent. It's possible that women who take hormone replacement therapy, or NSAIDs, are in some way different from women who do not, which will bias the findings. Determination of the clinical implications of these findings would most appropriately be resolved in future trials, designed to address the question of interest.

**Additional Information.:**

Please access these Web sites via the online version of this summary at http://dx.doi.org/10.1371/journal.pmed.0040157.

- Resources from the US National Institutes of Health on menopausal hormone therapy, including links to information about the Women's Health Initiative trials, information about managing menopausal symptoms, and more
- Resources from the US National Institutes of Health (MedlinePlus) about heart disease in women
- Information from NHS Direct, the UK National Health Service, about hormone replacement therapy
- The UK General Practice Research Database is the database utilized in this article
- Wikipedia entry on nonsteroidal anti-inflammatory drugs (NSAIDs) (note: Wikipedia is an internet encyclopedia anyone can edit)

## Introduction

Premenopausal women are less susceptible to myocardial infarction (MI) and stroke than are males of the same age group, an advantage that is lost after menopause [1]. However, the mechanism by which female gender affords cardioprotection is unclear. Despite the cardiovascular advantage of premenopausal women, it has been difficult to identify a cardioprotective effect of hormone therapy (HT) in postmenopausal women [2]. We recently found in mice that estrogen acts via its ERα receptor to up-regulate COX-2–dependent prostacyclin (PGI_2_) formation which, acting via its receptor (the I prostanoid receptor) constrains both platelet activation and oxidant stress. Indeed, deletion of the I prostanoid receptor undermined substantially the vascular benefit of estrogen therapy in ovariectomized mice rendered prone to atherogenesis by deletion of the low-density lipoprotein receptor [3].

These observations prompted us to address the possibility that the failure to detect a benefit from estrogen might be partly attributable to a pharmacodynamic interaction with inhibitors of COX-2, the major source of PGI_2_ in vivo. Sufficient information is currently unavailable to address this question for selective inhibitors of COX-2. However, traditional (t) nonsteroidal anti-inflammatory drugs (NSAIDs), such as ibuprofen, variably inhibit both COX-1 and COX-2, unlike low-dose aspirin, which targets preferentially, albeit not exclusively, COX-1 [4,5]. The objective of the current study is to provide a preliminary estimate of the interaction, in a general population setting, between tNSAIDs and HT on the occurrence of acute myocardial infarction (AMI) and death from coronary heart disease (CHD).

## Methods

### Source of Data

The General Practice Research Database contains computerized medical information entered systematically by general practitioners (GPs) in the UK and sent anonymously to the Medicines and Healthcare Products Regulatory Agency [6]. The information recorded includes demographic data, outpatient clinical diagnoses, consultant referrals, hospital admissions, and prescriptions. More than 90% of all referrals present in the manual records in GPs' offices are entered into computer files with a code that reflects the clinical diagnosis. Prescriptions are generated directly from the GP's computer and entered into the patient's computerized file. The term “tNSAIDs” included the following agents: aceclofenac, acemetacin, azapropazone, diclofenac, diflunisal, etodolac, fenbufen, fenoprofen, flurbiprofen, ibuprofen, indomethacin, ketoprofen, ketorolac, mefenamic acid, meloxican, nabumetone, naproxen, piroxicam, sulindac, tenoxicam, and tiaprofenic. There was insufficient information to explore with precision the interaction at the individual tNSAID level. tNSAIDs did not include aspirin nor selective COX-2 inhibitors. The latter were introduced in the UK in late 2000 when the study period ended. The three most widely used tNSAIDs (diclofenac, ibuprofen, and naproxen) accounted for 75% of all tNSAID use.

### Study Design

We recently evaluated the association between tNSAIDs and acute MI [7], where more details of the study population are provided. The results were compatible with the absence of a major beneficial or detrimental effect of tNSAIDs on the risk of MI. The study design and methods are described in detail in this paper. In brief, we identified all individuals aged 50–84 y on 1 January 1997. We started following up patients from the first day after that date, once they met the criteria of at least two years' enrolment with the GP and one year since the first computerized prescription of any drug. That date was designated as their start date. We excluded patients with a diagnosis of cancer before the start date. We followed up all study cohort members from the start date until the earliest occurrence of one of the following endpoints: a first recorded diagnosis of MI, cancer, death, age 85 y, date of last practice data collection, or 31 December 2000. We reviewed computerized profiles of all patients with a code of MI as well as all deaths. We used the adapted international standardized diagnostic criteria to consider an individual as having an AMI or dying from CHD [8,9]. Ultimately, 4,795 patients were included and the date of admission to the hospital or the date of death was treated as index date. All of the women were included as AMI individuals (*n* = 1,673) in the present study. We considered as fatal those individuals who died within the first 30 d after the occurrence of AMI and patients who died from CHD before reaching the hospital. Therefore in this study, designed a priori, AMI includes, if not otherwise stated, both fatal and nonfatal outcomes in the participants. A random date within the study period was generated for each of the study cohort members. All individuals with a random date included in their period of observation (from study entry to end of follow-up) were eligible as controls. A group of 20,000 control participants, frequency-matched by age, sex, and calendar year, was randomly sampled among with the study cohort. We applied to the controls the same computer-based exclusion criteria as we applied to AMI indviduals, using each participant's random date as his or her index date. For the present study, we included all women as control participants (7,005 controls).

Information on coronary risk factors, comorbidities, and drug utilization was obtained from the database. Hypertension, diabetes, hyperlipidemia, smoking, cardiovascular disease, arthritis, and recent anemia were considered present when these specific diagnoses were registered in the database before the index date. History of CHD was defined by the presence of MI and/or angina. Cerebrovascular disease was defined by the presence of ischemic or hemorrhagic stroke. Body mass index, expressed in kg/m^2^, was calculated from the registered height and weight. Alcohol intake was used as directly registered by the GP. We also elicited the participants' use of the health services (visits to the GP, specialist referrals, and hospital admissions) in the two years before the index date.

A list of all medications containing estrogens and/or progestogens recommended for HT and available in the UK during the study period was extracted from the British National Formulary. These drugs were grouped into the following regimens: (a) oral oestrogens; (b) transdermal oestradiol; (c) oestradiol implant; and (d) tibolone. In addition, oral oestrogens and transdermal oestradiol were classified as opposed (59% of all HT) or unopposed (41% of all HT) depending on whether a progestin was supplied along with oestrogens. Exposure to HT was classified into three groups: “current users” when the supply of the most recent prescription lasted until index date or ended in the year before the index date; “past users” when it ended more than one year before the index date; and “nonusers” when there was no recorded use before the index date. When current use was restricted to those exposed only in the month before the index date, the estimate for the interaction was slightly higher. There was considerable—73%—overlap in the women considered current users by the two definitions. The definition of current use for all other studied drugs, including tNSAIDs, was use of the drug in the month before the index date.

### Statistical Analyses

A nested case-control analysis was performed to estimate the effect of HT as well as the interaction between HT and tNSAIDs on the risk of MI. We calculated the odds ratio (OR) and 95% confidence intervals (CIs) of MI using unconditional logistic regression. All estimates of risk were adjusted for age, smoking, hypertension, diabetes, obesity, hypercholesterolemia, ischemic heart disease, use of low-dose aspirin, and antihypertensive drugs.

## Results

**Figure 1:**
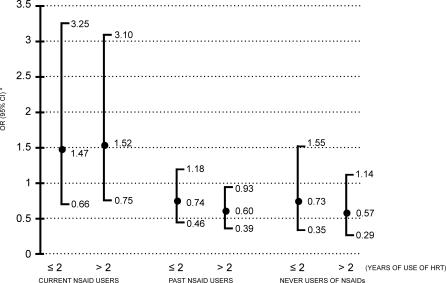
Acute MI and Current Use of HT by Duration, Stratified for Use of tNSAIDs *ORs adjusted for age, history of smoking, hypertension, diabetes, obesity, hypercholesterolemia, ischemic heart disease, use of low-dose aspirin, and antihypertensive drugs. The estimates of OR associated with current use of HT were calculated using non-use of HT as reference group in each of the three NSAID strata presented.

Overall, current use of HT was associated with a reduced risk of MI with an OR of 0.78 (95% CI 0.61–1.00) (Table 1). The corresponding OR among long-term users of HT, defined as exceeding 2 y, was 0.72 (95% CI 0.52–0.99). However, this suggestion of cardioprotection from HT disappeared (Figure 1) in women who were taking tNSAIDS concomitantly (OR 1.50; 95% CI 0.85–2.64). If tNSAIDs had not been used in the preceding month (combining the past users and never users of NSAIDs), use of HT resulted in an OR of 0.66 (95% CI 0.50–0.88). When we performed a standard test of interaction, the level of significance attained was *p* = 0.0012. The figure also shows that these estimates of risk did not vary by duration of HT. The interaction was apparent in women taking both opposed and unopposed estrogens, with ORs of 1.39 (95% CI 0.65–2.95) and 1.86 (95% CI 0.79–4.42), respectively. Also, the interaction between HT and tNSAIDs was already evident within months of starting the tNSAID and was sustained, irrespective of the duration of tNSAID therapy or HT (Table 2). Menopausal status is often lacking in the General Practice Research Database. A secondary analysis restricted to women older than 55 attained similar results: use of HT in the absence of tNSAIDs resulted in an OR of 0.68 (95% CI 0.50–0.93) among women in this age group. Another subset analysis restricted to women free of CHD yielded an OR of 1.57 (95% CI 0.78–3.18) among long-term users of HT taking tNSAIDs concomitantly.

**Table 1:**
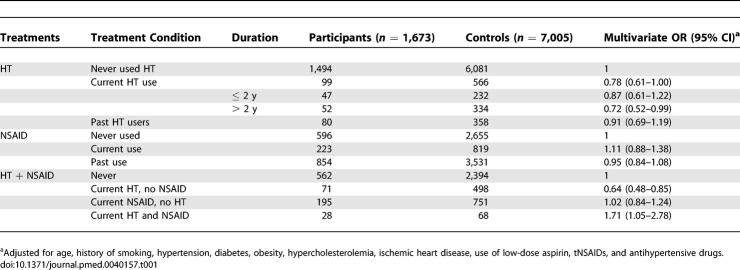
Risk of Acute MI in Users of HT by Duration, Users of NSAIDs, and Concomitant Users of HT and NSAIDs

**Table 2:**
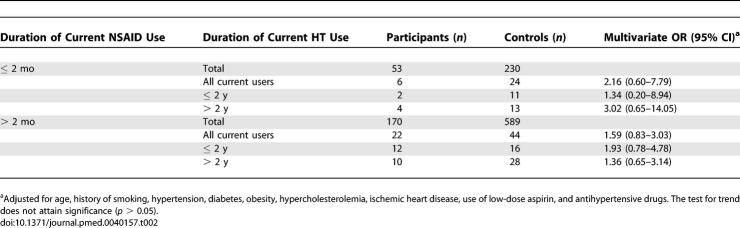
Risk of Acute MI in Users of HT by Duration Stratified According to Duration of Current tNSAID Use

Prompted by these results, we sought the interaction in a secondary analysis of current and past HT users and in HT nonusers for patients on low- and high-dose aspirin, antihypertensives, antidepressants, and gastric acid–suppressing drugs—a further 15 comparisons. Clearly, it is possible that a significant result might be attained amongst such multiple comparisons by chance. Concomitant use of aspirin at 75mg/d, which preferentially targets COX-1 for inhibition, was associated with an OR of 0.71 (95% CI 0.26–1.94) approximating that in aspirin nonusers (OR 0.77; 95% CI 0.59–1.00). However, the OR of a MI rose to 1.41 (95% CI 0.47–4.22) amongst users of HT with concomitant doses of aspirin 150 mg/d and above, where coincident inhibition of COX-2 should be more pronounced. We replicated these analyses adjusting for health services utilization, and the results were materially unchanged (unpublished data). We investigated whether this interaction was specific to tNSAIDs, and found that commonly used drugs, such as antihypertensive drugs, acid-suppressing drugs (proton pump inhibitors and/or histamine II antagonists), and tricyclic antidepressants failed to reveal an interaction with HT (unpublished data). Thus, the interaction between COX inhibitors and HT appeared to be specific. Also, when we performed a sensitivity analysis using a one-month time window instead of one year to define current use of HT, the OR of HT in women who were taking tNSAIDS concomitantly further rose to 1.85 (95% CI 1.01–3.41). Although we adjusted for matching factors (age, sex, calendar time) in our analysis and for a series of known cardiovascular risk factors, an unrecognized and therefore unmeasured factor that strongly related both to concomitant use of HT and NSAIDs on one hand, and to the risk of MI on the other might theoretically contribute to our results.

## Discussion

The results of this study raise the possibility that concomitant medication with NSAIDs might undermine a cardioprotective effect of HT in perimenopausal women. Mechanistically, this might relate particularly to inhibition of COX-2. However, the present study pertained to tNSAIDs, which inhibit COX-2, but vary in their comcomitant inhibtion of COX-1 and did not include NSAIDs designed to attain specific inhibition of COX-2.

Both the pattern of incidence and the manifestation of vascular disease differ between women and men. Premenopausal women exhibit a diminished burden of vascular disease compared with males of similar age; this difference is eclipsed after menopause [1]. Furthermore, the excess incidence of MI over stroke is more pronounced in men than in women [10]. Aspirin is effective in the primary prevention of MI in men [11,12] and in the primary prevention of stroke in women [10], possibly reflecting a true difference in drug response between the sexes. However, when the absolute incidence of all events is greater in trials of secondary prevention, aspirin detectably reduces the incidence of both MI and thrombotic stroke in both genders [13].

The present study raises the possibility of a drug–drug interaction with cardiovascular implications involving COX inhibitors. Much controversy has surrounded the putative cardioprotective effects of HT reported in observational studies [14]. Indeed, the only randomized controlled trial (RCT)—the Women's Health Initiative (WHI) study—failed to support this contention overall. While observational studies have most frequently found an approximate 30% reduction in CHD amongst users of HT, there was an increase in risk of CHD in HT users in the first years of follow-up in WHI. Some of the observational studies have limitations, including healthy user bias, compliance bias, inclusion of a small number of new initiators of HT, and choice of the reference group [15]. Yet, even after adjustment for these methodological issues, the summary estimate associated with HT use reflects a risk reduction of close to 20% [15].

Generally, when the results of observational studies are in conflict with an RCT, the balance of evidence favors the latter. While observational studies can control for recognized variables, they are vulnerable to an imbalanced distribution of unrecognized variables of relevance to the outcome of the study. On the other hand, WHI was also affected by some methodological constraints. The first was a differential detection bias [16]. Close to 40% of those allocated to HT, but only 7% of those allocated to placebo, were unblinded at some point during the RCT. The second was the gradual loss of the baseline comparability between the HT and placebo arms over time (average 5 y). The third was the limited power to estimate with precision the apparent protective effect appearing after long-term HT exposure. However, perhaps an even more important difference between WHI and other RCTs of HT and the observational studies is the age of the women investigated. For example, two-thirds of users in the WHI were aged 60 y or above at the beginning of the RCT [17], whereas only about one-fifth of users in the general UK population were in that age range in the year 2000 [18]. As summarized recently by Mendelsohn and Karas [19], the age at which women initiate HT is likely to be critical; evidence from animal models suggest that beneficial effects of HT in the cardiovascular system would only be anticipated if it is initiated before the development of advanced atherosclerosis. The potential importance of this difference has been highlighted by a recent analysis of the WHI data based on patient age and years since menopause. Women who initiated HT close to menopause tended to have a reduced risk of coronary heart disease while risk appeared to increase among women starting HT more distant from the menopause [20]. Our studies in mice revealed that loss of PGI_2_ impacted particularly on initiation and early development of atherosclerosis [3]. Thus, an age-dependent cardiovascular benefit from HT might both reconcile the apparent discrepancy between the observational studies and WHI and be consistent with a pharmacodynamic interaction between HT and NSAIDs. Furthermore, recent studies in mice indicate that progesterone, used in combination with estrogen in WHI, antagonizes the vasoprotective effect of estrogen on antioxidant enzyme and function [21]—the apparent mechanism disrupted by inhibition of COX-2–dependent PGI_2_ [3]. Thus, we felt it most appropriate to seek preliminary evidence for the clinical importance of these observations in studies of NSAID use in younger women, in whom observational data suggest consistently that long-term HT may confer cardiovascular benefit. Clearly, additional RCTs will be necessary to address residual controversies relating to cardioprotection from HT.

We have reported that estrogen dependent atheroprotection in mice depends in substantial part on COX-2 derived PGI_2_. Scant information is presently available concerning the combined use of specific COX–2 inhibitors and HT. However, we examined a priori a database for an interaction between tNSAIDs and HT. Generally, the coincident, time-dependent inhibition of platelet COX-1–derived thromboxane A_2_ might be expected, if anything, to mitigate the impact of COX-2 inhibition by the tNSAIDs examined in this study [22]. We do not have sufficient information to determine the effects of individual tNSAIDs. However, it is pharmacologically plausible that within this category are drugs that favor inhibition of COX-2 (e.g. diclofenac), inhibit both COX enzymes coincidentally over time (e.g. ibuprofen), afford cardioprotection in some individuals (e.g. naproxen), or interact ( e.g. ibuprofen and naproxen) to undermine cardioprotection from low-dose aspirin [5,23]. Future studies—ideally an RCT—will determine whether these properties differentially influence an interaction with HT. Similarly, we have insufficient information to permit a formal subanalysis of individual hormonal preparations or route of drug delivery, although a differential effect is not evident from what data are available. Information on dosage, adequacy, and sustenance of COX inhibition by patients taking aspirin or NSAIDs are unavailable in the WHI, as was any objective confirmation of either consumption of or abstention from such readily available COX inhibitors. Thus, we cannot project the likely impact of our findings on the failure to detect a cardioprotective effect of HT in WHI. However, if this interaction is indeed clinically meaningful, we would expect it to only partially explain such an effect. Concurrent treatment with tNSAIDs obscured roughly one-fifth of the benefit of HT in the present observational study. Finally, given the failure to detect a cardiovascular benefit of HT in the WHI before stratification for patient age or time since the menopause, the failure to detect an interaction with NSAIDs undermining such a benefit [24] is unsurprising.

In summary, these observations, based on small numbers, are provocative rather than conclusive and are not intended to guide clinical practice, but rather to prompt additional research. However, they raise the possibility that coincident inhibition of COX-2 by tNSAIDs may undermine cardioprotective effects of HT in peri- and postmenopausal women. If evidence consistent with this interaction were obtained in other larger epidemiological datasets, the possibility could fruitfully be addressed directly in randomized controlled clinical trials.

## Abbreviations

AMI: acute myocardial infarction
CHD: coronary heart disease
CI: confidence interval, COX, cyclooxygenase
GP: general practitioner
HT: hormone therapy
MI: myocardial infarction
NSAID: nonsteroidal anti-inflammatory drugs
OR: odds ratio
PGI_2_: prostacyclin
RCT: randomized controlled trial
tNSAID: traditional nonsteroidal anti-inflammatory drugs
WHI: Women's Health Initiative
